# Coronavirus Host Genomics Study: South Africa (COVIGen-SA)

**DOI:** 10.1155/2022/7405349

**Published:** 2022-10-06

**Authors:** Andrew K. May, Heather Seymour, Harriet Etheredge, Heather Maher, Marta C. Nunes, Shabir A. Madhi, Simiso M. Sokhela, W. D. Francois Venter, Neil Martinson, Firdaus Nabeemeeah, Cheryl Cohen, Jocelyn Moyes, Sibongile Walaza, Stefano Tempia, Jackie Kleynhans, Anne von Gottberg, Jeremy Nel, Halima Dawood, Ebrahim Variava, Stephen Tollman, Kathleen Kahn, Kobus Herbst, Emily B. Wong, Caroline T. Tiemessen, Alex van Blydenstein, Lyle Murray, Michelle Venter, June Fabian, Michéle Ramsay

**Affiliations:** ^1^Sydney Brenner Institute for Molecular Bioscience (SBIMB), Faculty of Health Sciences, University of the Witwatersrand, Johannesburg, South Africa; ^2^Division of Human Genetics, National Health Laboratory Service and School of Pathology, Faculty of Health Sciences, University of the Witwatersrand, Johannesburg, South Africa; ^3^Wits Donald Gordon Medical Centre, School of Clinical Medicine, Faculty of Health Sciences, University of the Witwatersrand, Johannesburg, South Africa; ^4^Steve Biko Centre for Bioethics, School of Clinical Medicine, Faculty of Health Sciences, University of the Witwatersrand, Johannesburg, South Africa; ^5^South African Medical Research Council, Vaccines and Infectious Diseases Analytics (VIDA) Research Unit, School of Pathology, Faculty of Health Sciences, University of the Witwatersrand, Johannesburg, South Africa; ^6^Department of Science and Technology, National Research Foundation, South African Research Chair Initiative in Vaccine Preventable Diseases, Faculty of Health Sciences, University of the Witwatersrand, Johannesburg, South Africa; ^7^Ezintsha, Wits Health Consortium, University of the Witwatersrand, Johannesburg, South Africa; ^8^Perinatal HIV Research Unit, University of the Witwatersrand, Johannesburg, South Africa; ^9^John Hopkins University Center for TB Research, Baltimore, MD, USA; ^10^Centre for Respiratory Diseases and Meningitis, National Institute for Communicable Diseases and School of Pathology, Faculty of Health Sciences, University of the Witwatersrand, Johannesburg, South Africa; ^11^Division of Infectious Diseases, Department of Internal Medicine, Chris Hani Baragwanath Academic Hospital, University of the Witwatersrand, Johannesburg, South Africa; ^12^Department of Medicine, Greys Hospital, Pietermaritzburg, South Africa; ^13^Caprisa University of KwaZulu-Natal, Durban, South Africa; ^14^Klerksdorp-Tshepong Hospital Complex, Klerksdorp, and Department of Medicine, University of the Witwatersrand, Johannesburg, South Africa; ^15^MRC/Wits Rural Public Health and Health Transitions Unit (Agincourt), School of Public Health, Faculty of Health Sciences, University of the Witwatersrand, Johannesburg, South Africa; ^16^Africa Health Research Institute, Durban, KwaZulu-Natal, South Africa; ^17^Division of Infectious Diseases, University of Alabama at Birmingham, Birmingham, AL, USA; ^18^Centre for HIV and STIs, National Institute for Communicable Diseases, and Faculty of Health Sciences, University of Witwatersrand, Johannesburg, South Africa; ^19^Division of Pulmonology, Department of Internal Medicine, Chris Hani Baragwanath Academic Hospital, University of the Witwatersrand, Johannesburg, South Africa

## Abstract

Host genetic factors are known to modify the susceptibility, severity, and outcomes of COVID-19 and vary across populations. However, continental Africans are yet to be adequately represented in such studies despite the importance of genetic factors in understanding Africa's response to the pandemic. We describe the development of a research resource for coronavirus host genomics studies in South Africa known as COVIGen-SA—a multicollaborator strategic partnership designed to provide harmonised demographic, clinical, and genetic information specific to Black South Africans with COVID-19. Over 2,000 participants have been recruited to date. Preliminary results on 1,354 SARS-CoV-2 positive participants from four participating studies showed that 64.7% were female, 333 had severe disease, and 329 were people living with HIV. Through this resource, we aim to provide insights into host genetic factors relevant to African-ancestry populations, using both genome-wide association testing and targeted sequencing of important genomic loci. This project will promote and enhance partnerships, build skills, and develop resources needed to address the COVID-19 burden and associated risk factors in South African communities.

## 1. Introduction

The severe acute respiratory syndrome coronavirus 2 (SARS-CoV-2) has resulted in over 540 million infections and over six million deaths since the first outbreak was detected in December 2019 [1]. Although rapid improvements in disease prevention and management have occurred with increased uptake of vaccines [2] and immunomodulation and oxygen therapy strategies for those hospitalised [3, 4], the pandemic remains a substantial worldwide problem that is anticipated to persist for the foreseeable future.

At the outset of the COVID-19 pandemic, there were fears that Africa would suffer the worst of the disease's impact [5,6]. Virus transmission was forecast to be unmanageably high due to limited healthcare infrastructure, few human health resources, and poor socioeconomic circumstances [5, 7–9], leading to understandable concerns about the impact of COVID-19, particularly among people living with tuberculosis (TB), HIV, and/or comorbid non-communicable diseases [10–12]. Preliminary statistics from Western countries suggested that individuals of Asian, Black, and Hispanic ethnicity were at greater risk of COVID-related death [13], mostly due to poor socioeconomic indicators in minoritised, underserved populations [7, 14]. Initial predictions for Africa were dire, with 70 million infections and 3 million deaths forecasted by June 2020 [8].

**Figure 1:**
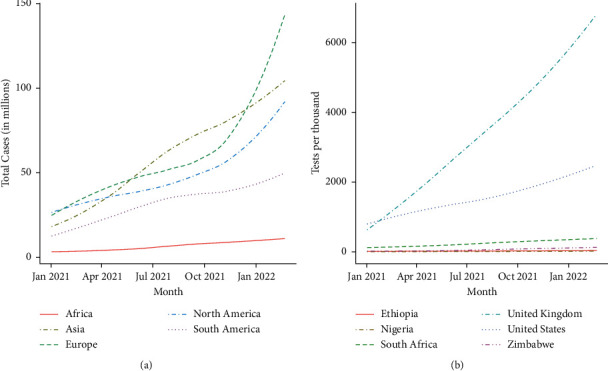
COVID-19 case numbers and tests per thousand across different regions: (a) confirmed case numbers on the African continent remain low in comparison to other continents despite early predictions that African countries would struggle the most to maintain infection control and (b) however, testing per thousand individuals in selected African countries is a fraction of those conducted in, for example, the United States and the United Kingdom. Data sourced from [19, 20].

However, the true impact of SARS-CoV-2 in Africa has been difficult to gauge as epidemiological surveillance infrastructure and widespread access to screening and testing have been extremely limited (Figure 1) and vary from region to region [15]. Routine death reporting occurs in a handful of African countries, and among these, coverage is often incomplete [16]. Nevertheless, some have speculated that the relative youth of African populations [17], their increased exposure to infectious pathogens including other coronaviruses [18], and prior vaccination (such as with the Bacille Calmette–Guerin vaccine), might serve as protective factors against SARS-CoV-2 [7].

Based on the clinical heterogeneity of COVID-19 [26] and reports of fulminant illness and death among young patients in good health [24, 27], host genetic factors have been suspected to moderate disease susceptibility, severity, and outcomes [28]. Several global initiatives have fostered collaboration between human geneticists, enabling rapid sample collection and data analysis. For example, the COVID Human Genetic Effort (HGE) [29] is concerned with identifying single gene inborn errors of immunity, which are likely to be rare but of large effect size, while the COVID-19 host genetics initiative (HGI) [21] seeks to understand the role of common, small-to-medium effect size variants throughout the human genome. The most recent meta-analysis from the COVID HGI [22], incorporating over 940,000 participants, supports at least 15 independent association signals across the genome, which together implicate close to 50 genes in modifying disease severity and/or susceptibility to infection. Priority candidates among this list of genes include *TYK2, PPP1r151, ABO, FOXP4, IFNAR2, DPP9, CXCR6, LZTFL1,* and *TMEM65*, which either have biological plausibility or contain coding sequence variants in strong linkage disequilibrium with a lead signal. Other strong candidates include *ACE2* and *TMPRSS2*, which both regulate the entry of SARS-CoV-2 into cells [23]. Meanwhile, studies investigating single gene inborn errors have identified toll-like receptor 3 (*TLR3*), *TLR7* [24], and type I interferon immunity in predisposing individuals to critical COVID-19 illness [14, 25].

The portability of disease-associated genetic signals across different geographies and ethnicities is limited [30, 31], with examples relevant to COVID-19. The lead-associated variant implicating the *FOXP4* gene (primarily expressed in the airway epithelium) has a high frequency of the effect-allele in Middle Eastern and East Asian communities (40%) but is of less significance in European populations, where the effect-allele frequency is capped at only 3% [22]. The level of expression of the candidate gene *ACE2* is known to decline from Europeans to Asians [7], possibly explaining the greater burden of COVID-19 in Italy and Spain. Additionally, *ACE2* is a target of ACE inhibitor class drugs, to which Africans generally respond poorly compared to other ethnicities, suggesting the presence of genetic variation that may also impact COVID-19 [6]. Globally, the frequency of an insertion/deletion variant in the related *ACE1* gene varies widely, with the COVID-19 high-risk deletion allele more common outside East Asia [7, 32]. Currently, the leading genetic risk factor for severe COVID-19 among European-ancestry populations is a 50 kb haplotype, on chromosome 3, reported to be introgressed from Neanderthals [33]. This haplotype has not been found to confer equivalent risk in Indian communities [34] and is near-absent among continental Africans [33]. Meanwhile, a disease protective splice variant in *OAS1* occurs more frequently in African-ancestry individuals (58%) compared to Europeans (32%; [35]), suggesting that genetic risk profiling for COVID-19 is likely different among Africans.

To date, there has been little representation of continental African populations in COVID-19 host genomic studies. In a recent meta-analysis from the COVID HGI, only 5% of 48,714 participants were of African ancestry [22]. This lack of African-specific data poses serious limitations to efforts aimed at diminishing health disparities between people from different ethnic backgrounds and between high and low-income countries [36]. Understanding host genetic factors promises to improve COVID-19 disease risk profiling [23] and could provide attractive targets for therapeutic drug design [37].

Motivated by these concerns and responding to calls from others emphasising the need for better inclusion of non-European participants in COVID-19 research [23], we established the coronavirus host genomics study: South Africa (COVIGen-SA). COVIGen-SA is a strategic collaboration between multiple study partners designed to promote and facilitate COVID-related research that is specific to Black South Africans. The primary aim of COVIGen-SA is to explore host genetics, providing insights into factors that moderate COVID-19 susceptibility, severity, and outcomes in a continental African population. The secondary aim is to build a research resource of demographic, clinical, and genetic variables (and associated DNA samples) from Black South Africans that can be accessed to answer research questions. Ultimately, COVIGen-SA is designed to provide a foundation for new cross-disciplinary partnerships and leverage skills and infrastructure needed to bolster COVID-19 host genetic research in Africa.

## 2. Methods

### 2.1. Experimental Design and Scope

#### 2.1.1. Participant Recruitment Model

To be eligible for enrollment into COVIGen-SA, potential participants may be of any sex and can reside in either rural or urban settings. Black African individuals over the age of 18 are prioritised for inclusion, but participants of other ethnicities and ages are increasingly included as the study expands. Note that “Black African” is a South African government-utilised racial category encompassing all individuals of African ancestry. While Black South Africans are of mostly south-eastern Bantu-speaking descent [38], their genetic composition may be variably admixed with other African and non-African ethnicities [39].

**Figure 2:**
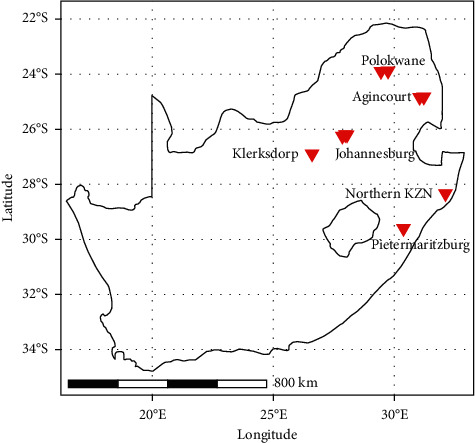
Study sites for COVIGen-SA. Participants for COVIGen-SA are currently being recruited from 10 different sites, in and around areas including Johannesburg, Polokwane, Bushbuckridge, Klerksdorp, Pietermaritzburg, and Northern KwaZulu Natal (KZN).

To maximise recruitment efficiency and minimise costs, we have multiple partners across the country (Figure 2; Table 1), all of whom are involved in separate research projects with specific inclusion/exclusion criteria in which participants are being actively recruited and health-related data collected. Some of these partner studies are directly concerned with medical aspects of COVID-19, while others perform PCR tests for SARS-CoV-2 infection as part of their study's inclusion/exclusion and/or follow-up criteria. This approach capitalises on existing infrastructure and resources for participant recruitment and limits research fatigue in participants by integrating consent and sample collection into existing participation sessions. From consenting participants, data and samples are captured and stored.

At first, we seek to enroll 5,000 Black South African participants across three clinical categories of COVID-19 disease, defined as follows: (i) critical COVID-19 illness—hospitalised cases requiring supplemental oxygen, ventilatory, or other organ support and/or who have died as a result of COVID-19; (ii) moderate to severe illness—hospitalised cases not requiring ventilatory or other organ support; and (iii) mild or asymptomatic disease—PCR-confirmed SARS-CoV-2 infection but asymptomatic or mild symptoms. These categories were selected to align closely with those used by the COVID HGI [22]. Participant recruitment will continue beyond 5,000 individuals should resources permit.

For comparison, we will use a population control sample comprised 5,000 Black South African individuals from the Africa Wits-INDEPTH Partnership for Genomic Studies (AWI-Gen), for whom genome-wide genotyping data is already available [43, 44]. AWI-Gen is an NIH-funded Collaborative Centre of the Human Heredity and Health in Africa Consortium [43] and has participants from four African countries. We note that the use of population control is a limitation of the study design, given the unavoidable presence of a subset of control individuals who may develop severe COVID-19 illness once exposed [22]. However, the use of population control has yielded robust findings in other studies [22, 37] and is considered a valid strategy.

#### 2.1.2. Data Capture and Storage

A study-specific instrument was designed to capture variables pertinent to the investigation of COVID-19 (Table 2). The instrument comprises three broad sections, including a demographic section (sex, age, self-reported ethnicity, living conditions), a general health section (past/current comorbidities and medications), and a COVID-19 section (diagnosis, symptoms, and disease outcomes). For new recruitments, instrument responses are recorded directly into a REDCap database. For partner studies collecting similar variables, we import the harmonised data into this database. Participant DNA is extracted from 6-10 ml venous blood samples in EDTA, stored at 4°C. Within one week the EDTA tubes are collected from study sites and centrifuged to separate out the buffy coats, which are frozen at -80°C until DNA extraction is required. DNA is extracted using an automated method performed on the Qiasymphony SP platform. DNA samples are then stored in an ethics-approved biobank (clearance certificate number: BEC20200401), located at the Sydney Brenner Institute for Molecular Bioscience (SBIMB). Genome-wide genotyping of DNA samples will be conducted in batches, as funding permits, using the H3Africa genotyping array [45]. The H3Africa array is custom-designed, incorporating 2.3 million SNP markers enriched for common variants in African genomes, and was previously used to genotype AWI-Gen participants who will form the control sample for COVIGen-SA.

#### 2.1.3. Analysis Strategy

**Figure 3:**
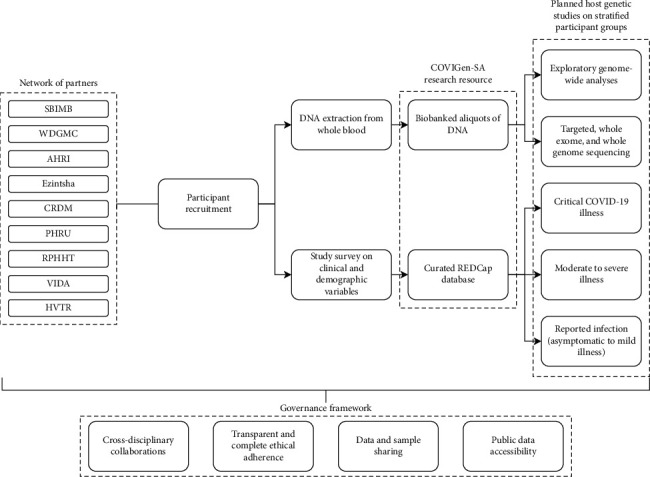
Overview of the COVIGen-SA research resource and planned host genetic studies. COVIGen-SA is based on a governance framework that promotes cross-disciplinary collaboration and transparent data and sample sharing that is ethically approved and legally compliant. In addition to the SBIMB and WDGMC, seven partners have joined the study to date, all contributing to a unified research resource that will facilitate host genetic and other COVID-related studies. The project data will be made available to improve the representation of continental Africans in public data sets. SBIMB: Sydney Brenner Institute for Molecular Bioscience, WDGMC: Wits Donald Gordon Medical Centre, AHRI: Africa Health Research Institute, CRDM: Centre for Respiratory Disease and Meningitis, National Institute for Communicable Diseases, PHRU: Perinatal HIV Research unit, RPHHT: Rural Public Health and Health Transitions Research Unit, (Agincourt) VIDA: Vaccines and Infectious Diseases Analytics Unit, and HVTR: HIV Vaccine Translational Research Entity.

During and after the establishment of the research resource, we plan to conduct several investigations into host genetic factors. These include, but are not limited to, genome-wide association analysis using different phenotypic categories, haplotype analysis, and bioinformatic fine-mapping. Further data collected from targeted sequencing, whole exome, and whole genome sequencing will facilitate additional investigations such as novel variant discovery and the identification of signals for selection. More nuanced investigations, such as sex-specific and burden analyses [46], will also be explored. An overview of the project design is shown in Figure 3.

#### 2.1.4. Study Coordination and Ethical Considerations

**Figure 4:**
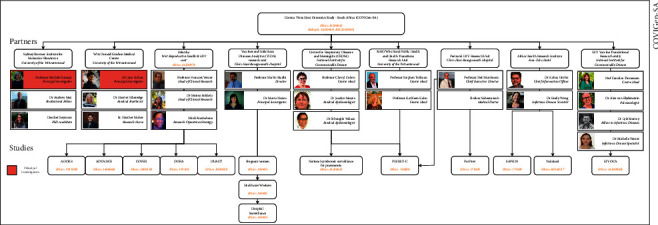
An organisational chart of the COVIGen-SA project. COVIGen-SA currently incorporates five institutions and seven study partners. Each partner is engaged in one or several independent studies from which eligible participants were recruited for the COVIGen-SA study. Each study has ethical clearance, while each partnership is also covered by an ethically approved agreement.

COVIGen-SA is headed by Professor Mich'ele Ramsay (principal investigator) and Dr. June Fabian (Co-PI) and is jointly based at the Wits Sydney Brenner Institute for Molecular Bioscience and the Wits Donald Gordon Medical Centre (WDGMC). The project leverages the strengths of these institutes, benefitting from the sample and data storage capabilities at the SBIMB Biobank, as well as the institute's track record for bioinformatic analysis of genomic data [39], and the ethical, clinical research expertise, and experience of the WDGMC. The organisational structure of COVIGen-SA is summarised in Figure 4. COVIGen-SA has received ethical clearance from the University of Witwatersrand Human Research Ethics Committee—Medical (HREC [M]; clearance number M200642). For each additional project linked to COVIGen- SA, an amendment is submitted to include or link a substudy or new cohort. Existing study-specific ethics clearance certificate numbers are provided in Figure 4. Participation requirements are minimal, including additional consenting processes (including specific consent for genetic studies and sharing of data and specimens) and, in some cases, an additional 6–10 ml EDTA venous blood draw. We administer the COVIGen-SA instrument only in situations where the partner study is not already collecting similar variables. Throughout recruitment, COVID-19-related guidelines are followed to protect both researchers and participants from infection. COVIGen-SA demonstrates a collective commitment to furthering our understanding of COVID-19 through robust scientific collaboration and information sharing.

### 2.2. Data Quality Control and Availability

Preprocessing of clinical and demographic data will focus on mitigating missing data and determining whether such data are missing completely at random, missing at random, or not missing at random [47]. In line with Anderson et al. [48], preprocessing of genomic data will be conducted at both a per-individual and per-marker level prior to removing individuals and/or SNP markers from the data set. Individuals will be earmarked for removal if (a) sex information is discordant between genotype data and self-reported sex and (b) genotyping or heterozygosity rates are outliers. Depending on the nature of the downstream analyses, individuals may be removed if cryptic relatedness is detected (i.e., removing one individual from a pair sharing an identity by descent score >0.1875) and/or participant ancestry is divergent from the majority of cases based on principal component analysis. SNP markers will be removed should they have excessive missing genotype information (a call rate < 95%), a low minor allele frequency (< 0.05, depending on the sample size and analysis being performed), or substantial deviation from Hardy–Weinberg equilibrium (P < 0.001). To maximise coverage across the genome, SNP genotype imputation will be leveraged using an appropriate reference panel. Raw and preprocessed data will be made available upon request and subject to a data-transfer agreement and appropriate ethical clearance. DNA samples will be available, conditional on ethical clearance, a material transfer agreement, and availability of DNA. 

## 3. Results

**Figure 5:**
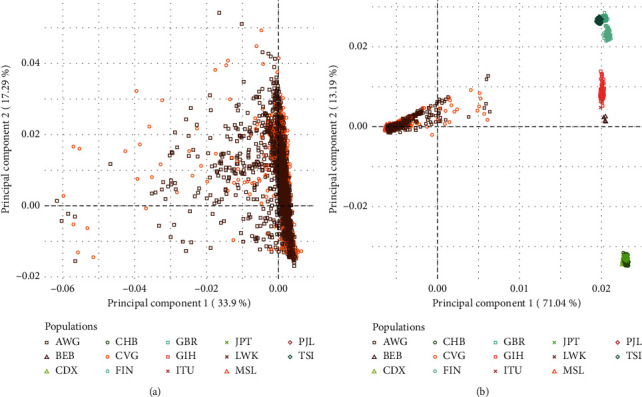
Preliminary PCA plots of COVIGen-SA data. (a) Initial genotyping results for 503 COVIGen-SA (CVG, case) participants were merged with data from 5,139 Black South African AWI-Gen (AWG, control) participants. Participants from both studies overlapped substantially, suggesting common ancestry, but some individuals map further from the centre of the cluster, reflecting the considerable genetic variation of Black South Africans and possible minor admixture components from other ancestries. (b) Case and control data were then merged with publicly available 1,000 Genomes Project data for select populations across the globe (*n* = 2,504). The first principal component separated continental African ethnicities from others. The second principal component then separated out non-African ethnicities. AWG: AWI-Gen (controls); BEB: Bengali in Bangladesh; CDX: Chinese Dai in Xishuangbanna, China; CHB: Han Chinese in Beijing, China; CVG: COVIGen-SA (cases); FIN: Finnish in Finland; GBR: British in England and Scotland; GIH: Gujarati Indian in Houston, TX; ITU: Indian Telugu in the UK; JPT: Japanese in Tokyo, Japan; LWK: Luhya in Webuye, Kenya; MSL, Mende in Sierra Leone; PJL: Punjabi in Lahore, Pakistan; and TSI: Toscani in Italia.

COVIGen-SA currently includes collaborative efforts across five institutions and seven study partners that together enable potential participant recruitment across 14 different studies and 10 recruitment sites, situated across 5 of the 9 provinces in South Africa (Figure 2). Participant recruitment commenced in October 2020. At the time of writing, data and samples had been collected from over 2,000 participants (from six of 14 studies), of whom 1,354 are reported here (Table 3). In line with demographics for the African continent, the majority of participants are younger than 40 years of age. Both rural- and urban-dwelling individuals are represented, distinguished most notably by the number of residents per household, which was larger amongst rural dwellers (e.g. in the PHIRST-C cohort; Kruskal-Wallis test statistic = 493.29, adjusted *p*<0.01). Documented comorbidities are diverse, ranging from high cholesterol and hypertension to renal disease and cancer. Given the high disease burden in South Africa and our partnership with HIV-focused research groups, COVIGen-SA is anticipated to include a high proportion of HIV comorbid participants, with 329 (24.30%) such participants enrolled to date. COVID-19 symptom severity also varies substantially across enrolled participants, although severely affected (i.e. hospitalised, and requiring either supplementary oxygen or mechanical ventilation) individuals remain underrepresented at present (n = 333, 24.59%). Efforts continue to prioritise the recruitment of severely affected individuals who are more likely to harbour large effect size genetic variants modifying COVID-19 severity. In the first genotyping batch of 576 participants, 73 were removed during preliminary quality control procedures (four due to sample failure and 69 due to divergent ancestry). Remaining participant genotypes were merged with AWI-Gen (control) [40, 41] and 1000 Genomes Project data [49] and principal component analysis conducted (Figure 5) using PLINK (version 1.90b6.21) [50, 51], R (version 4.1.0) [52], RStudio (version 1.4.1103) [53], and the ggplot2 package (version 3.35) [54]. Case and control participants clustered together (Figure 5(a)), suggesting common ancestry, but Black South African samples were substantially more dispersed compared to other ethnicities (Figure 5(b)), in line with the known magnitude of genetic variation for continental Africans [39, 55].

## 4. Discussion

### 4.1. Anticipated Impact

Continental Africans have the highest genetic diversity compared to all other ethnicities [55, 56] and thus harbour individual variants and patterns of variation not observed elsewhere [57, 58]. Deeper and more extensive genetic profiling is imperative to understanding (and ultimately improving) health outcomes for African-ancestry individuals [59, 60]. As evidence gathers to implicate host genetic factors in COVID-19 outcomes, it is reasonable to assume that novel associations and/or variants, possibly private to African genomes, may help in understanding the impact of the pandemic in Africa [55]. Our preliminary principal component analyses (Figure 5) reiterate the known genetic diversity of self-identified Black South Africans, for whom no COVID-19 host genomic research has been completed to date. In addition to unparalleled genetic variation, our current participants already represent a heterogeneous array of demographic and health-related backgrounds. All considered, our sample holds the substantial potential to reveal novel insights into COVID-19. The COVIGen-SA research resource has thus been designed to facilitate host genetic, and possibly other, explorations into this unique sample. While the primary motivation is to help contribute towards alleviating the disease burden in this specific population, we also anticipate the knowledge will have relevance to other African ethnicities and will provide additional opportunity for documenting and understanding medically relevant genetic variation in Africa more broadly. Furthermore, we expect that COVIGen-SA will have meaningful outcomes regarding scientific capacity development in South Africa, high-impact publications, and collaborations that cross multiple disciplines. Future manuscripts will centre on genetic investigations, guided by the results of our early GWAS findings. These publications will promote awareness and improve literacy regarding the importance of genetic factors in COVID-19 host response and the need for African-specific research.

### 4.2. Study Governance

The COVID-19 pandemic has fundamentally altered the global status quo, introducing substantial challenges necessitating collaboration and cooperation on an unprecedented scale [61]. Scientific research, in particular, has relied on increased collaboration, particularly interdisciplinary, to respond to the pandemic [62] and to stay abreast of the evolving lineages of SARS-CoV-2 [63], most recently illustrated by the emergence of the Omicron variant, which dominated the fourth wave of infection [64].

The COVIGen-SA study has developed several interdisciplinary partnerships that should maximise the impact of the project and assist in overcoming the logistic and financial limitations to scientific research imposed by COVID-19. This is particularly relevant in the developing country context of Africa, where infrastructural challenges may restrict the degree to which research priorities can be addressed [15], further entrenching health disparities laid bare by the pandemic [61]. Cooperative research endeavors are thus doubly important if Africa is to keep pace with the rest of the world. The requisite urgency of responding to COVID-19 has resulted in substantial upward pressure on the ethics and regulatory infrastructure of research internationally, yet the imperatives of ethical research remain, and these need to be carefully considered and addressed. COVIGen-SA has been built upon an ethics and regulatory framework that seeks to maximise participant protection and respond to the unique research challenges presented by the pandemic (Figure 4). In South Africa, this framework has been developed within a vacuum, as no guidelines for ethics in pandemic research were previously available. However, the National Human Research Ethics Committee (NHREC) has recently released a draft Pandemic Research Ethics guideline for public comment. The ethics framework of COVIGen-SA already largely conforms to this guideline but will be revised as guidance emerges. It is similarly critical that we do not sacrifice academic integrity for the sake of “speed science” (a problem brought into sharper focus due to the pandemic) [65], which runs the risk of erroneous claims. To this end, we aim to share the COVIGen-SA research resource as widely as possible so that it may be assessed and validated by other research groups. We have developed a data protection and sharing framework that will enable us to do this both locally and internationally, according to the relevant data privacy legislation and ethical principles. Ultimately, we hope that the post-COVID emphasis on scientific collaboration becomes a guiding principle for research governance, especially in Africa, and ideally in international research that better represents the African continent.

### 4.3. Challenges and Limitations

Although supported by a strong foundation of partnerships, several key challenges remain for the COVIGen-SA study. Of these, funding is the most pressing concern. Participant recruitment and sample ascertainment costs have been kept to a minimum by aligning our recruitment with the studies of our partners, but sample storage and genotyping expenses remain a considerable strain on available funds. Secondly, we have struggled to recruit individuals severely affected by COVID-19 for a variety of reasons. In the main, the infrastructural and logistic shortcomings of South Africa's health system reduce our ability to identify the full scope of severely affected COVID-19 patients. The majority of our partner studies have similarly noted a lack of severely affected patients in their cohorts. Although the limited number of severe patients might speak to the resilience of Black South Africans against SARS-CoV-2 infection [35], the estimates of excess deaths in the country [66] suggest that these individuals may not be receiving the timeous intervention and appropriate support at clinics and hospitals. Lastly, based on the preliminary principal component analysis, the genetic diversity among COVIGen-SA participants is substantial. Such diversity may dilute our ability to find clear genetic association signals, but next-generation sequencing efforts are poised to reveal novel variations that may shed further light on the genetic aetiology of COVID-19.

Logistical challenges aside, we foresee some limitations to the current design of the study. Despite our numerous partnerships and recruitment sites, our sample size is likely to remain small compared to other host genomic studies (e.g., [35]), reducing our power to detect/replicate smaller effect size associations. Furthermore, our phenotypic database will comprise data from several independent studies, in which some data points may be missing when collating and harmonising the final data set. These data will have been collected over various waves of COVID-19 infection (driven by different viral variants) and at various stages of vaccine rollout, which could potentially undermine the representativeness of our sample and may introduce confounding effects we could not have anticipated at the start of the pandemic. However, these limitations are balanced against the uniqueness of our sample, both in terms of genetic and clinical diversity, which remains a particular strength of African-centric research.

## 5. Conclusion

Accurately determining the burden of COVID-19 in South Africa and other African countries remains challenging and important in the context of health planning. While vaccine rollout begins to alleviate some of the pressure of the pandemic, significant impetus remains to develop improved therapeutic approaches to COVID-19, especially as the SARS-CoV-2 virus mutates and evolves.

Research attention directed at this problem should be inclusive of communities worldwide if meaningful progress in reducing health inequality is to be made. COVIGen-SA represents our attempt not only to contribute to the fight against a global pandemic but also to serve an underrepresented ethnicity in genetic research. We envisage the project as a unifying framework that brings together otherwise disconnected efforts to study the host genomics of COVID-19 in South Africa. We continue to scan the horizon for further collaboration and funding opportunities and look forward to maximising the anticipated outcomes and impact of the project.

## Figures and Tables

**Table 1:** A brief overview of COVIGen-SA partner studies.

Partner	Project names	Description of project	Participant inclusion criteria	Catchment area	Self-identified ethnicity	Target sample size
*A*	ADORE	A single-arm, phase 3, pilot study investigating the efficacy of doravirine in adults living with HIV experiencing virological failure on first-line EFV-based ART with NNRTI resistance.	PLHIV, 18 years or older with confirmed first generation NNRTI resistance on first-line EFV-based ART.	Clinics and hospitals, Johannesburg, Gauteng province (urban)	Black African	25
	ADVANCE	A randomised, phase 3 non-inferiority study of DTG + TAF + FTC compared with DTG + TDF + FTC and EFV + TDF + FTC in patients infected with HIV-1 starting first-line ART.	PLHIV, ART-naïve, 12 years and older.	Clinics and hospitals, Johannesburg, Gauteng province (urban)	Black African	1,053
	COVER	A multicentre, randomised, open-label study of NTZ or SOF/DCV, compared to no pharmacological intervention for the prevention of COVID-19 disease in healthcare workers and inner city inhabitants at high risk of exposure to SARS-CoV-2.	People of 18 years and older at high risk of exposure to SARS-CoV-2, no existing symptoms of COVID-19, not vaccinated against COVID, emale participants—no intention to fall pregnant.	Clinics and hospitals, Johannesburg, Gauteng province (urban)	Black African	1,950 (25% enrolled)
	DORA	A single-arm, phase 3, pilot switch study, exploring the safety of doravirine-based first-line ART for women of reproductive potential living with HIV.	Females living with HIV, between 18 and 49 years old, on reliable contraception or not intending to fall pregnant, on first-line ART comprising EFV/TDF/FTC or DTG/TDF/3TC.	Clinics and hospitals, Johannesburg, Gauteng province (urban)	Black African	100
	DUACT	A randomised double-blinded study to assess the antiviral efficacy, safety, and tolerability of dual combination antiviral coronavirus therapy (ribavirin and NTZ) in ambulatory participants diagnosed with acute COVID-19.	People between 18 and 75 years old, diagnosed with SARS-CoV-2 infection, with at least one constitutional symptom (but not critically ill), with onset within 72 hours.	Clinics and hospitals, Johannesburg, Gauteng province (urban)	Black African	52 (65% enrolled)

*B*	Healthcare Workers	A study investigating the epidemiology of SARS-CoV-2 infection among healthcare workers from Chris Hani Baragwanath Academic Hospital.	Any healthcare worker from Chris Hani Baragwanath Hospital. All participants were enrolled in COVIGen-SA.	Soweto, Johannesburg, Gauteng province (urban)	Not specified	400
	Hospital surveillance	A study describing the burden and clinical characteristics of COVID-19-associated hospitalisations.	People of 18 years or older admitted to Chris Hani Baragwanath Hospital with a confirmed SARS-CoV-2 infection.	Soweto, Johannesburg, Gauteng province (urban)	Not specified	10,000
	Pregnant women	A study evaluating the association of SARS-CoV-2 infection during pregnancy and poor fetal outcomes.	Pregnant women or women in labour who are of 18 years or older attending Chris Hani Baragwanath Academic Hospital or Rahima Moosa Mother and child hospital with confirmed SARS-CoV-2 infection.	Soweto and Coronationville, Johannesburg, Gauteng province (urban)	Not specified	3,000

*C*	PHIRST-C	A prospective household study of SARS-CoV-2, influenza, and RSV community burden, transmission dynamics, and viral interaction in South Africa.	3 or more individuals living in a household, all willing to consent and be available for twice weekly testing.	SA/MRC wits Agincourt health and demographic surveillance system site, Bushbuckridge, Mpumalanga province (rural); Jouberton, Klerksdorp, north-west province (urban)	Majority Black African	600 at each site
	National surveillance	A prospective active surveillance programme for respiratory illness, including testing for SARS-CoV-2, influenza, RSV, and pertussis.	People of all ages fulfilling the clinical case definition of a severe respiratory illness.	Helen Joseph and Rahima Moosa Mother and child hospital, Johannesburg, Gauteng province (urban)	Any	400 per month for the duration of surveillance

*D*	Pot prev	A surveillance and observational study looking at the incidence of physician-diagnosed, community-acquired pneumonia in three provinces in South Africa.	People of 18 years and older with clinical and radiologically confirmed community-acquired pneumonia.	Soweto, Johannesburg, Gauteng province; Klerksdorp, North West province; Polokwane, Limpopo province (all sites are urban)	Black African, White, mixed ancestry	Soweto: 900 Klerksdorp: 900 Polokwane: 200
*E*	AHRI	A node of the South African population infrastructure network (SAPRIN) that supports four surveillance sites in South Africa. Vukuzazi is a population multiomics cohort within the AHRI node of SAPRIN that aimed to measure the prevalence and overlap of four diseases (HIV, TB, hypertension, and diabetes) in an HIV endemic population and collect biosamples to support research into the social, environmental, and biological drivers of health and disease.	SAPRIN: All household members resident within the boundaries of the respective surveillance areas (140,000 individuals). Vukuzazi: 18,000 individuals aged 15 and older—consented to genomic analysis of stored blood samples. Participants are screened for SARS-CoV-2, with positive cases enrolled in COVIGen-SA.	Northern KZN (rural), Agincourt Mpumalanga (rural)	Black African	SAPRIN: 140,000 Vukukazi cohort: 18,000

*F*	GIVOCA	A longitudinal observational study of adult SARS-CoV-2 RT-PCR confirmed hospitalised adults.	People hospitalised with SARS-CoV-2 infection, 18 years or older, with or without HIV.	Soweto and surrounding areas, Gauteng province (urban)	Black African	200

^*∗*^ *A*: ezintsha, *B*: vaccines and infectious diseases analytics research unit, *C*: centre for respiratory diseases and meningitis and the MRC/wits rural public health and health transitions research unit [40], *D*: Perinatal HIV Research Unit, *E*: African health research institute (AHRI) [41, 42], *F*: HIV vaccine translational research entity, 3TC: lamivudine, ART: antiretroviral treatment, DCV: daclatasvir, DTG: dolutegravir, EFV: efavirenz, FTC: emtricitabine, HIV: human immunodeficiency virus, NNRTI: non-nucleoside reverse transcriptase inhibitor, NTZ: nitazoxanide, PLHIV: people living with HIV, RSV: respiratory syncytial virus, SOF: sofosbuvir, TAF: tenofovir alafenamide, and TDF: tenofovir disoproxil.

**Table 2:** Demographic and clinical variables collected for COVIGen-SA.

Category	Variable
Demographics	Sex
	Ethnicity
	Age at collection
	Nearest town to current residence province of current residence
	Number of people living in current residence
	Number of rooms used for sleeping in current residence

Health information	Smoking behaviour
	Asthma
	Cancer
	Cardiovascular disease
	Diabetes mellitus
	HIV
	Hypertension
	Lung disease (excluding asthma)
	Pulmonary tuberculosis
	Renal disease
	Previous lung or other organ transplants

Medication/treatment (current)	Inhaled corticosteroids
	Chemotherapy, radiation, and/or cancer-related surgery
	Antiretrovirals
	ACE-inhibitors
	Oral steroids
	Tuberculosis treatment
	Dialysis
	Any other chronic medications

Anthropometric measurements	Height
	Weight
	Body mass index

COVID-19 diagnosis	Method of testing
	Date of diagnosis
	Pregnant at diagnosis

COVID-19 symptoms	Cough
	Fatigue/malaise
	Anosmia ageusia
	Shortness of breath
	Sore throat
	Other symptoms

COVID-19 outcomes	Did not seek/require healthcare
	Hospitalised
	Hospitalised—oxygen required
	Hospitalised—ICU
	Hospitalised—ventilation

**Table 3:** Demographic summary of COVIGen-SA SARS-CoV-2 positive participants recruited from four studies.

	Ezintsha	PHIRST-C	GIVOCA	VIDA	Total
SARS-CoV-2 positive cases	378	300	123	553	1,354
Sex (% female)	61.11	60.52	57.72	75.59	64.66
Age (standard deviation)	29.58 (11.10)	25.12 (20.53)	50.11 (12.31)	46.38 (16.76)	33.45 (20.18)
Household members (median)	3	6	4	4	5
Severely affected	0	2	123	208	333
HIV+ participants	18	143	62	106	329

See Table 1 for a description of each study.

## Data Availability

The data used to support this study can be obtained from the corresponding author upon request.
